# Limonin Alleviates Non-alcoholic Fatty Liver Disease by Reducing Lipid Accumulation, Suppressing Inflammation and Oxidative Stress

**DOI:** 10.3389/fphar.2021.801730

**Published:** 2022-01-03

**Authors:** Yunjia Li, Menghan Yang, Haiyan Lin, Weixin Yan, Guanghui Deng, Haixin Ye, Hao Shi, Chaofeng Wu, Guoliang Ma, Shu Xu, Qinxiang Tan, Zhuowei Gao, Lei Gao

**Affiliations:** ^1^ School of Traditional Chinese Medicine, Southern Medical University, Guangzhou, China; ^2^ Shenzhen Hospital, University of Chinese Academy of Sciences, Shenzhen, China; ^3^ The First Affiliated Hospital of Guangzhou University of Chinese Medicine, Guangzhou, China; ^4^ Shenzhen Hospital, Beijing University of Chinese Medicine, Shenzhen, China; ^5^ Shunde Hospital, Guangzhou University of Chinese Medicine, Foshan, China; ^6^ Guangdong Provincial Key Laboratory of Shock and Microcirculation, Southern Medical University, Guangzhou, China; ^7^ Zhujiang Hospital, Southern Medical University, Guangzhou, China

**Keywords:** limonin, inflammation, NAFLD (non alcoholic fatty liver disease), oxidative stress, zebrafish

## Abstract

Non-alcoholic fatty liver disease (NAFLD) is the most common cause of chronic liver disease and continues to rise in the worldwide. Limonin is a triterpenoid compound widely found in the fruits of citrus plants with a wide range of pharmacological effects, including anti-cancer, anti-inflammation, anti-viral, anti-oxidation and liver protection properties. However, the potential molecular mechanism of limonin on NAFLD in zebrafish remains unknown. In this study, zebrafish larvae were exposed to thioacetamide to establish an NAFLD model and the larvae were treated with limonin for 72 h simultaneously. The human liver cell line was stimulated with lipid mixture and meanwhile incubated with limonin for 24 h. The results showed that Limonin significantly reduced the accumulation of lipid droplets in the liver and down-regulated the levels of lipogenic transcription factors FASN and SREBP1 in NAFLD. Limonin suppressed macrophages infiltration and the down-regulated the relative expression levels of the pro-inflammatory factors IL-6, IL-1β and TNF-α secreted by macrophages. Besides, limonin could reversed the reduction of glutathione and the accumulation of reactive oxygen species through up-regulating NRF2/HO-1 signaling pathway in the liver. In conclusion, this study revealed that limonin has a protective effect on NAFLD due to its resistance to lipid deposition as well as antioxidant and anti-inflammatory actions.

## Introduction

Non-alcoholic fatty liver disease (NAFLD) is now considered to be the most common liver disease in the world. It includes a wide range of conditions, from simple steatosis, to non-alcoholic diseases like steatohepatitis, fibrosis, and ultimately leading to liver cirrhosis and hepatocellular carcinoma (Eslam et al., 2018; Friedman et al., 2018). The global prevalence of NAFLD is estimated to be 25% and continues to rise during the global obesity epidemic (Mundi et al., 2020). The pathological progression of NAFLD temporarily follows a “multiple-hits” process, including steatosis, lipotoxicity and inflammation (Cobbina and Akhlaghi, 2017). Normally, fatty acids are stored in adipose tissue in the form of triglycerides, but mistakenly transferred from their main storage sites to ectopic sites such as bone and liver tissue during NAFLD (Fabbrini et al., 2009). And pro-inflammatory cytokines induced by steatosis contribute to the recruitment and activation of liver macrophages, which in turn mediate the development of non-alcoholic steatohepatitis (NASH) (Anderson and Borlak, 2008). The accumulation of lipids in the liver can lead to lipotoxicity and dysfunction of mitochondrial oxidation, leading to the production of reactive oxygen species (ROS). Oxidative stress in fatty liver disease has been considered to be the third attack that ultimately leads to liver cell death (Tiniakos et al., 2010). Therefore, the progress of NAFLD is closely related to the steatosis, oxidative stress and inflammation. New treatment strategies need to be determined and validated to treat NAFLD. Recently, natural compounds seem to have attracted much attention due to their effective roles in the treatment of liver diseases.

Limonin belongs to the tetracyclic triterpenoids and is usually derived from Rutaceae plants. Limonin can be isolated from many traditional Chinese botanical drugs and citrus fruits, including *Citrus reticulata* Blanco (*Rutaceae, Citri reticulatae pericarpium*)*, Citrus aurantium* L. (*Rutaceae, Aurantii fructus*)*, Coptis chinensis* Franch (*Ranunculaceae, Coptidis rhizoma*)*, Phellodendron chinense* C.K.Schneid (*Rutaceae, phellodendri chinenses cortex*) and *Citrus bergamia* Risso (*Rutaceae, Citri sarcodactylis fructus*) (Tundis et al., 2014; Fan et al., 2019). Recent studies have shown that limonin had a wide range of pharmacological effects, including anti-cancer, anti-inflammatory and analgesic, antibacterial and antiviral, anti-oxidant and reduce cholesterol, etc. (Gualdani et al., 2016; Fan et al., 2019). The extensive pharmacological effects of limonin have attracted widespread attention. However, the role and molecular mechanism of limonin in NAFLD have not been studied. Therefore, it is necessary to clarify the pharmacological mechanism of limonin on NAFLD and provide a scientific basis for clinical treatment of liver diseases.

Zebrafish has become a new animal model for biomedical research and drug discovery due to its easy genetic manipulation, high reproductive capacity, external fertilization and transparent embryos in recent years (Lessman, 2011) (Tonelli et al., 2020). Zebrafish can detect complete liver function at 3 days after fertilization (Zhou et al., 2019). There are many kinds of cell fluorescent labeling zebrafish, which can quickly visualize the physiological and pathological processes such as the composition, function, signal transduction and response to injury of liver cells (Goessling and Sadler, 2015). Therefore, zebrafish is a suitable model for exploring the effects of natural medicines on NAFLD based on high-throughput capabilities.

## Materials and Methods

### Antibodies and Reagents

**FIGURE 1:**
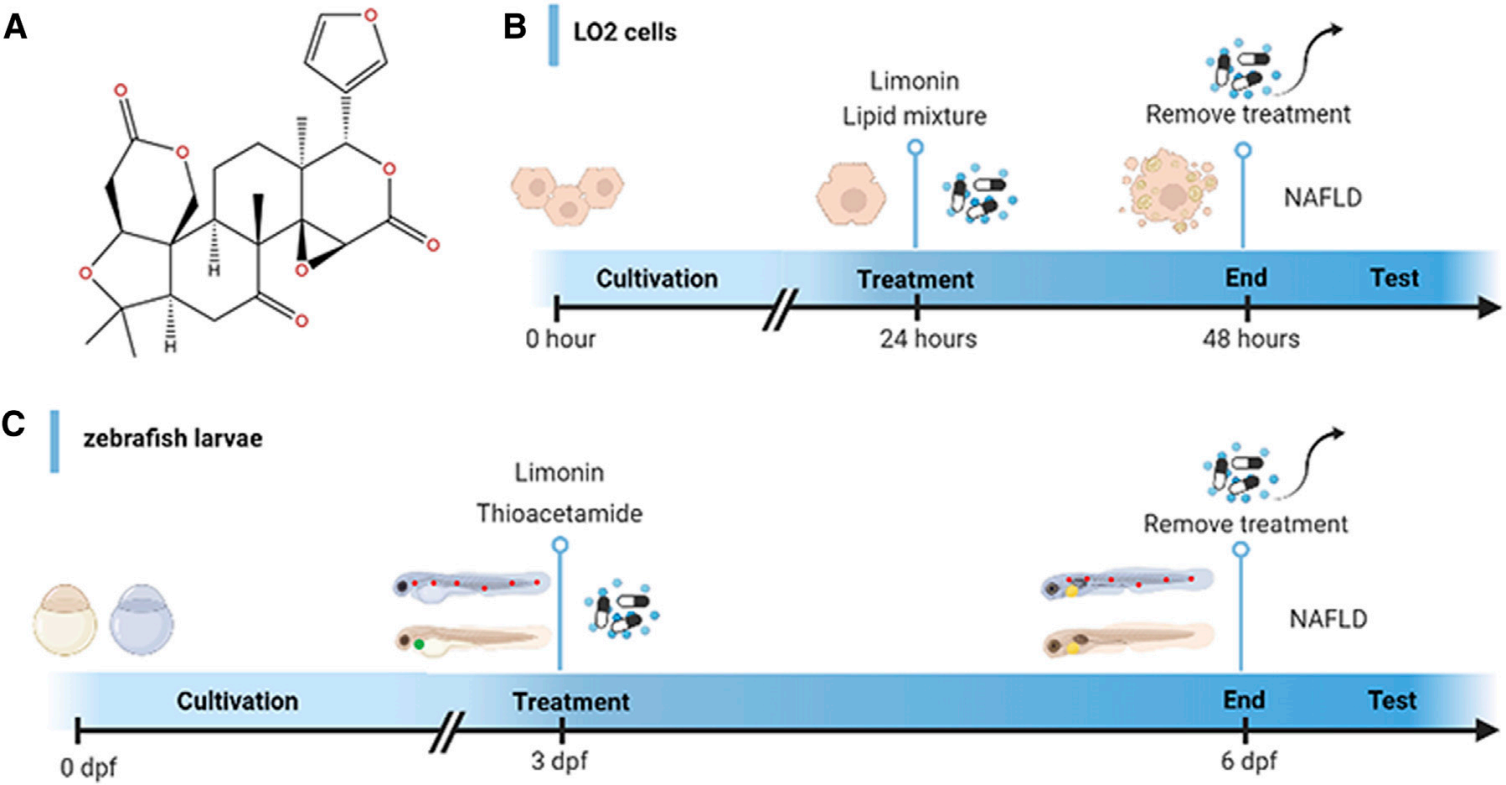
Design of experimental process. **(A)** Chemical structure of limonin. **(B)** Schematic diagram of drug treatment for LO2 cells. **(C)** Schematic diagram of drug treatment for zebrafish.

Limonin Analytical Standard (CAS Number:1180-71-8; C26H30O8) with purity (HPLC) ≥ 98.0% was purchased from Shanghai yuanye Bio-Technology Co., Ltd (Shanghai, China). Thioacetamide (CAS Number: 62-55-5; CH3CSNH2) was obtained from Sigma (United States). The chemical structure of limonin is presented in Figure 1A. Anti-SREBP1 (rabbit, 1:1000, ab28481), anti-TNF-α (rabbit, 1:100, ab1793) and anti-HO-1 (rabbit, 1:1000/1:500, ab13243) antibodies were purchased from Abcam (Cambridge, United Kingdom). Anti-FASN (rabbit, 1:1000, 3180S), anti-GAPDH (rabbit, 1:1000, 2118S), Goat anti-rabbit IgG (1:2000, 7074S), Alexa Fluor594-conjugated goat anti-rabbit IgG (1:250, 8889S) and Alexa Fluor488-conjugated goat anti-rabbit IgG (1:250, 4412S) antibodies were obtained from Cell Signaling Technology (United States). Anti-NRF2 (rabbit, 1:1000/1:200, 16396-1-AP) was obtained from proteintech (United States).

### Zebrafish Maintenance and Treatment

The zebrafish came from the Key Laboratory of Drug Screening for Zebrafish Models and Human Diseases, Southern Medical University. Wild-type zebrafish (AB strain), liver-specific eGFP transgenic zebrafish (Tg (lfabp10α:eGFP)), macrophage-specific red fluorescent protein expression transgenic line (Tg (mpeg1:dsred)) were used in this study. Zebrafish were kept in the light:dark period for 14:10 h at 28°C. Zebrafish fertilized embryos developed in egg water at 28.5°C (0.8 mg/L methylene blue). All zebrafish experimental procedures were approved by the Institutional Animal Care and Use Committee of Southern Medical University.

Zebrafish larvae at 3 days post fertilization (dpf) were randomly divided into five groups: the control group (raised in fish water), model group (treated with 0.4 mg/ml thioacetamide (TAA)), limonin group (limonin dissolved in dimethyl sulfoxide (DMSO)): treated with 0.4 mg/ml TAA and limonin simultaneously. The final concentration of limonin in each group was 12.5, 25 and 50 μM. The zebrafish larvae were exposed for 72 h in a 6-well plate at a density of 30 per hole. The experimental process is shown in Figure 1C.

### Cell Culture and Treatment

Human fetal hepatocyte cell line (LO2) was cultured in DMEM containing 10% FBS, 100 units/ml streptomycin and 100 mg/ml penicillin (Gibco, Carlsbad, CA, United States) in a humidified atmosphere of 37°C, 95% air and 5% CO2. The cells were randomly divided into five groups: control group, model group and limonin groups treated with corresponding concentrations. The cells were stimulated with lipid mixture 1 (L0288, Sigma, United States) for 24 h to establish an *in vitro* NAFLD model. The limonin intervention groups were incubated with 10% lipid mixture and limonin (10, 20 and 40 μM) for 24 h simultaneously. Cells were fixed in 4% paraformaldehyde or lysed after incubating for 24 h. The schematic diagram of drug treatment is shown in Figure 1B.

### Hematoxylin and Eosin Staining

Zebrafish larvae were fixed with 4% paraformaldehyde (PFA) at 4°C for 24 h, embedded in paraffin and cut into 4 μM sections. The specimens were deparaffinized, rehydrated, H&E stained, dehydrated, removed, and sealed for routine histological examination. Finally, a Nikon Eclipse Ni-U optical microscope (Nikon, Tokyo, Japan) was used to capture H&E sections.

### Oil Red O Staining

Whole-Fish: Fixed larvae were washed with phosphate buffered saline (PBS) for 5 min, and then penetrated by 20, 40, 80 and 100% 1,2-propanediol (Sigma, United States) each for 15 min. Then stained with 0.5% Oil Red O (Sigma, United States) at 65°C in the dark for 1 h. The zebrafish larvae were then incubated in 100% 1,2-propanediol at room temperature for 1 h, and eluted with 80, 40, and 20% 1,2-propanediol each for 10 min. Finally, larvae were imaged by an Olympus U-HGLGPS microscope (Tokyo, Japan).

Cryosections: Fixed zebrafish larvae were dehydrated in 30% sucrose at 4°C for 3 days. After being embedded in optimal cutting tissue (OCT) compound (Leica, Germany), larvae were cut into 14 μM sections. Frozen sections were washed with PBS to remove OCT, incubated in 100% 1, two propylene glycol for 5 min, then stained with 0.7% Oil Red O for 10 min at 60°C, finally eluted with 85% 1,2-propylene glycol and rinsed with PBS to keep the background clean. The slices were imaged with a Nikon Eclipse Ni-U optical microscope (Nikon, Tokyo, Japan).

Cell slides: Fixed cell slides were washed with PBS for 15 min, then incubated with 100% 1,2-propanediol for 10 min. After being stained with 0.7% Oil Red O and decontaminated with 85% 1,2-propanediol and PBS, the slides were imaged with Nikon Eclipse Ni-U optical microscope (Nikon, Tokyo, Japan).

### Nile Red Staining

The frozen sections of zebrafish larvae and cell slides were washed with PBS for 15 min. Cells and zebrafish sections were incubated with Nile Red solution for 10 min (Nile Red was dissolved in acetone at a concentration of 0.5 ug/ml), washed with PBS, stained with DAPI (Solarbio Life Science, China) for 5 min, and protected from light. The samples were imaged with Nikon Eclipse Ni-U fluorescence microscope (Nikon, Tokyo, Japan).

### Detection With Superoxide and Glutathione Probes

Used dihydroethidium (DHE, Beyotime, Shanghai, China) for ROS detection. A naphthylamide-sulfoxide-based fluorescent agent probe (NA-8, Future-Chase Biotechnology Co., Ltd.; FYRK-FP-01-003KY) was used to detect the content of glutathione (GSH). After being modeled with TAA for 72 h, zebrafish larvae were incubated with 10 μM DHE and NA-8 probes solution for 10 min at 28°C in the dark. Then the zebrafish larvae were transferred to the agarose plate and anesthesia with 0.2% tricaine (Sigma, United States) for captured. Nikon Eclipse Ni-U fluorescence microscope (Nikon, Tokyo) was used to image the distribution and intensity of the fluorescence of DHE and NA-8.

### Immunochemistry

Paraffin sections of zebrafish larvae were deparaffinized with xylene, rehydrated with ethanol, and sodium citrate was boiled for 10 min to recover the antigen, and cooled to room temperature. The sections were inactivated endogenous peroxidase in methanol with 3% H_2_O_2_ for 10 min and then washed with PBS. After being sealed with blocking buffer (5% normal goat serum and 0.1% Triton-X PBS solution) for 2 h, the sections were incubated with rabbit antibody or mouse antibody (diluted with 2.5% normal goat serum and 0.1% Triton-X PBS) at 4°C overnight. The next day, paraffin sections were washed three times with PBS, then incubated with goat anti-rabbit antibody for 2 h at room temperature. The sections were washed with PBS, followed by stained with DAB for less than 10 min and terminated with ice water. The samples were counter-stained with hematoxylin, dehydrated, and finally sealed with a neutral glue. The stained sections were photographed with an optical microscope (Nikon Eclipse Ni-U; Nikon, Tokyo, Japan).

### Immunofluorescence

Frozen sections of zebrafish larvae and cell slides were washed three times with PBS, soaked in methanol for 10 min and sealed with blocking buffer (5% normal goat serum and 0.1% Triton-X PBS solution) for 1 h at room temperature. The sections and cell slides were incubated with rabbit antibody (diluted with 2.5% normal goat serum and 0.1% Triton-X PBS) at 4°C overnight. After being washed with PBS, the samples were incubated with goat anti-rabbit antibody for 1 h at room temperature. Then washed with PBS and incubated with DAPI for 10 min in the dark. The samples were washed three times with PBS, followed by sealed with anti-fluorescence quencher and photographed with an optical microscope (Nikon Eclipse Ni-U; Nikon, Tokyo, Japan).

### Quantitative Real-Time PCR

TRIzol reagent was used to extract total RNA from 30 zebrafish larvae according to the instructions (15596018, Ambion™). Reverse transcription was performed in accordance with the instructions of the Takara kit (PrimeScript™ RT Reagent Kit with gDNA Eraser (Perfect Real Time). Then qPCR was performed on the LightCycler 96 instrument (Roche, Switzerland) with the TB Green^®^ Premix Ex Taq™ (Tli RNaseH Plus) kit. The ordered primers for each gene were obtained from BGI Tech Solutions (Beijing Liuhe) Co., Ltd. and were showed in Table 1. The β-actin gene was used as an internal reference gene and the CT value was calculated by the formula: 2^−ΔΔCt^.

**TABLE 1:** Primers used to quantify mRNA levels.

Gene	FP sequence (5′-3′)	RP sequence (5′-3′)
β-actin	ATG​GAT​GAG​GAA​ATC​GCT​GCC	CTC​CCT​GAT​GTC​TGG​GTC​GTC
FASN	GAG​AAA​GCT​TGC​CAA​ACA​GG	GAG​GGT​CTT​GCA​GGA​GAC​AG
IL-1β	TGG​ACT​TCG​CAG​CAC​AAA​ATG	CAC​TTC​ACG​CTC​TTG​GAT​GA
IL-6	AGA​CCG​CTG​CCT​GTC​TAA​AA	TTT​GAT​GTC​GTT​CAC​CAG​GA
gpx1a	AGG​CAC​AAC​AGT​CAG​GGA​TT	CAG​GAA​CGC​AAA​CAG​AGG​G
NRF2	AAGCAGACGGAGGAGGAG	GGAGGTGTTCAGGCAAGG

### Western Blot Analysis

Collected LO2 cells were washed three times with PBS, and lysed by cell lysate containing phosphatase inhibitor (Sigma), RIPA lysis buffer (Sigma) and protease inhibitor (Sigma) for 15 min, centrifuged, and then aspirated the supernatant for protein quantification and protein denaturation. A total of 30 μg protein was used for western blotting.

### Statistical Analysis

All statistical analysis was performed with GraphPad Prism Version 8.0 software and SPSS 20.0. The numerical results are expressed as mean ± standard deviation (SD). Statistical differences were evaluated by one-way ANOVA analysis of variance, followed by Tukey’s multiple comparisons test on dependent experimental designs. *p* value < 0.05 is considered statistically significant.

## Results

### Toxicology of Limonin in Zebrafish Larvae

**FIGURE 2:**
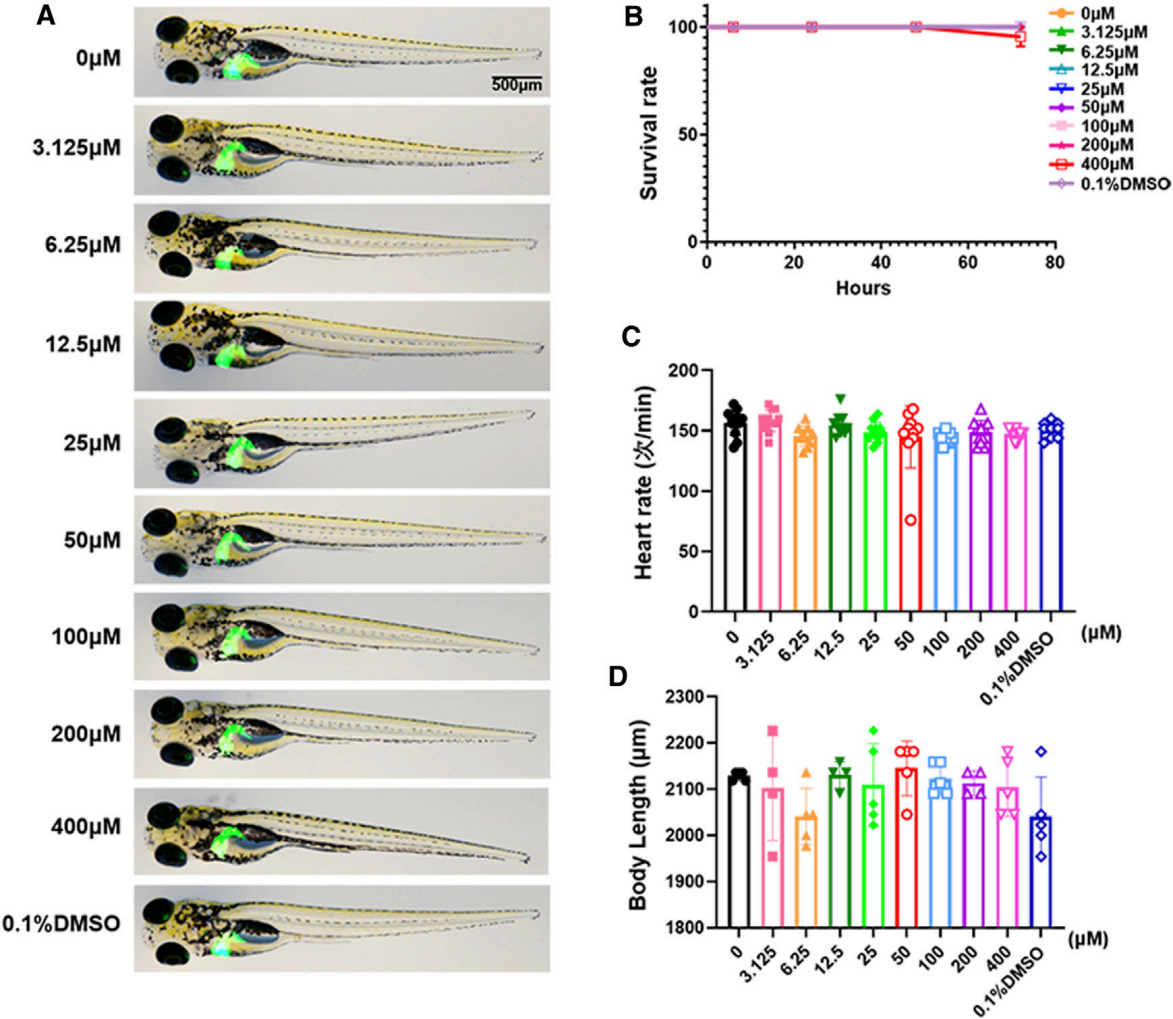
Toxicology of limonin in zebrafish larvae. **(A)** The effect of zebrafish larvae exposed to different concentrations of limonin for 72 h on zebrafish larval morphology and liver development (*n* = 6 per group) **(B)** The effect of different concentrations of limonin on the survival rate of zebrafish larvae (*n* = 22 per group). **(C)** Heart rate of zebrafish larvae exposed to different concentrations of limonin (*n* = 9–13 per group). **(D)** The body length of zebrafish larvae exposed to different concentrations of limonin (*n* = 4–6 per group). The data are shown as mean ± SD.

We used the survival rate, the heart rate, the body length and morphological changes to observe the toxicology of limonin in zebrafish larvae. Zebrafish larvae (3 dpf) were treated with limonin for 72 h. Zebrafish larvae had no morphological abnormalities were observed at the tested concentrations of limonin (Figure 2A), indicating that limonin has low drug toxicity to the morphology of zebrafish during development. 0-200 µM limonin had no effect on the survival of zebrafish larvae, but the survival rate of zebrafish larvae after treatment with 400 µM limonin for 72 h was 90% (Figure 2B). In the heart rate test and the body length test, different concentrations of limonin had no significant effect on the heart rate and body length of zebrafish larvae (Figures 2C,D). According to the results of drug toxicity studies, we found that limonin was safe and had low toxicity, and 12.5, 25, and 50 µM limonin were chose for further experiments.

### Limonin Alleviates TAA-Induced Hepatic Steatosis in Zebrafish

**FIGURE 3:**
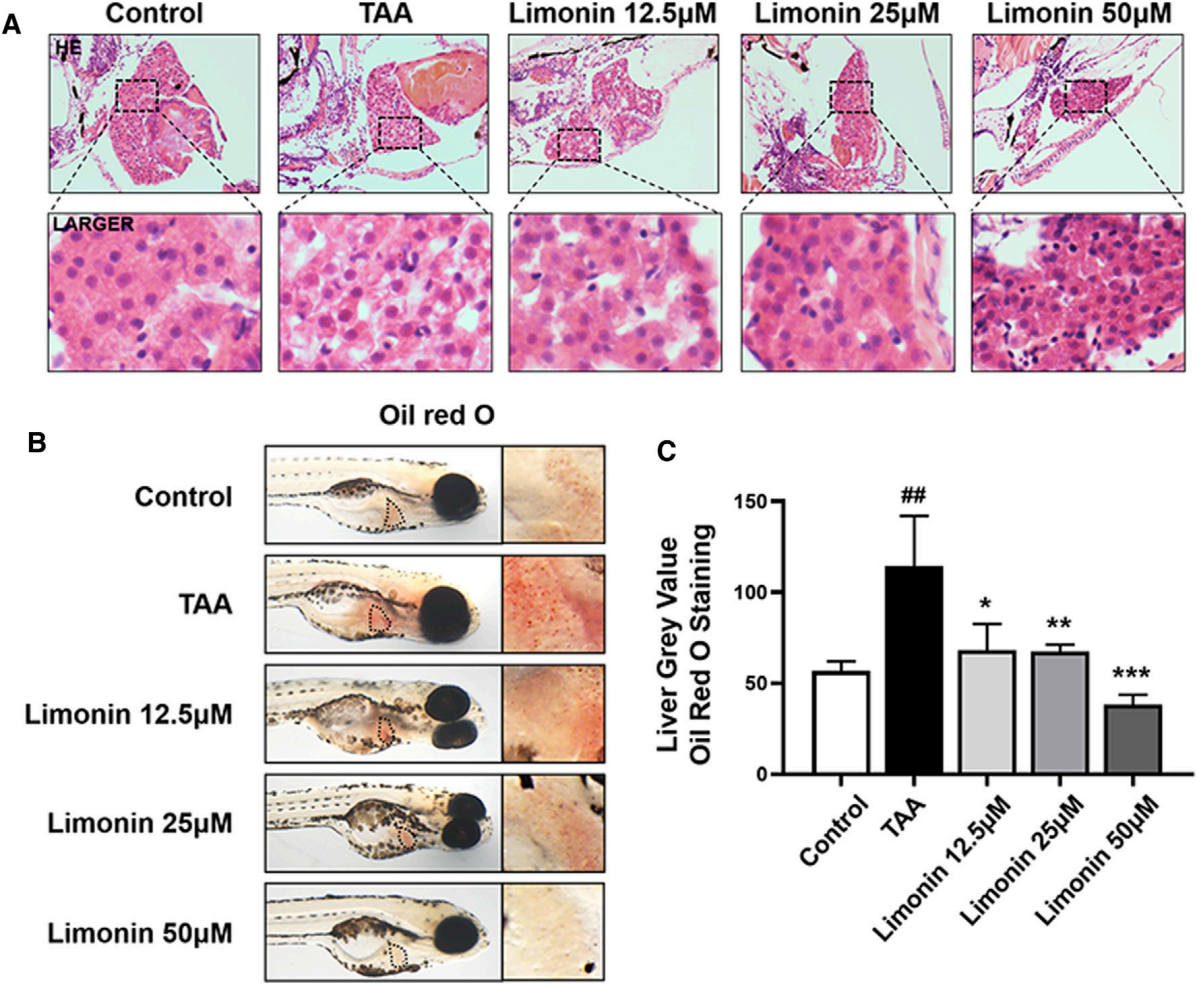
Limonin alleviated TAA-induced liver hepatic steatosis in zebrafish larvae. **(A)** H&E staining of zebrafish larvae. Figures are magnified as ×400 (*n* = 5–7 per group). **(B)** Whole fish staining with Oil Red O. Figures are magnified as ×50 (*n* = 3 per group). **(C)** Quantitative analysis of the liver gray value of Oil Red O using ImageJ software. Data are shown as the mean ± SD. #*p* < 0.05, ##*p* < 0.01, ###*p* < 0.001 vs control group; *p < 0.05, **p < 0.01, ***p < 0.001 vs TAA group.

**FIGURE 4:**
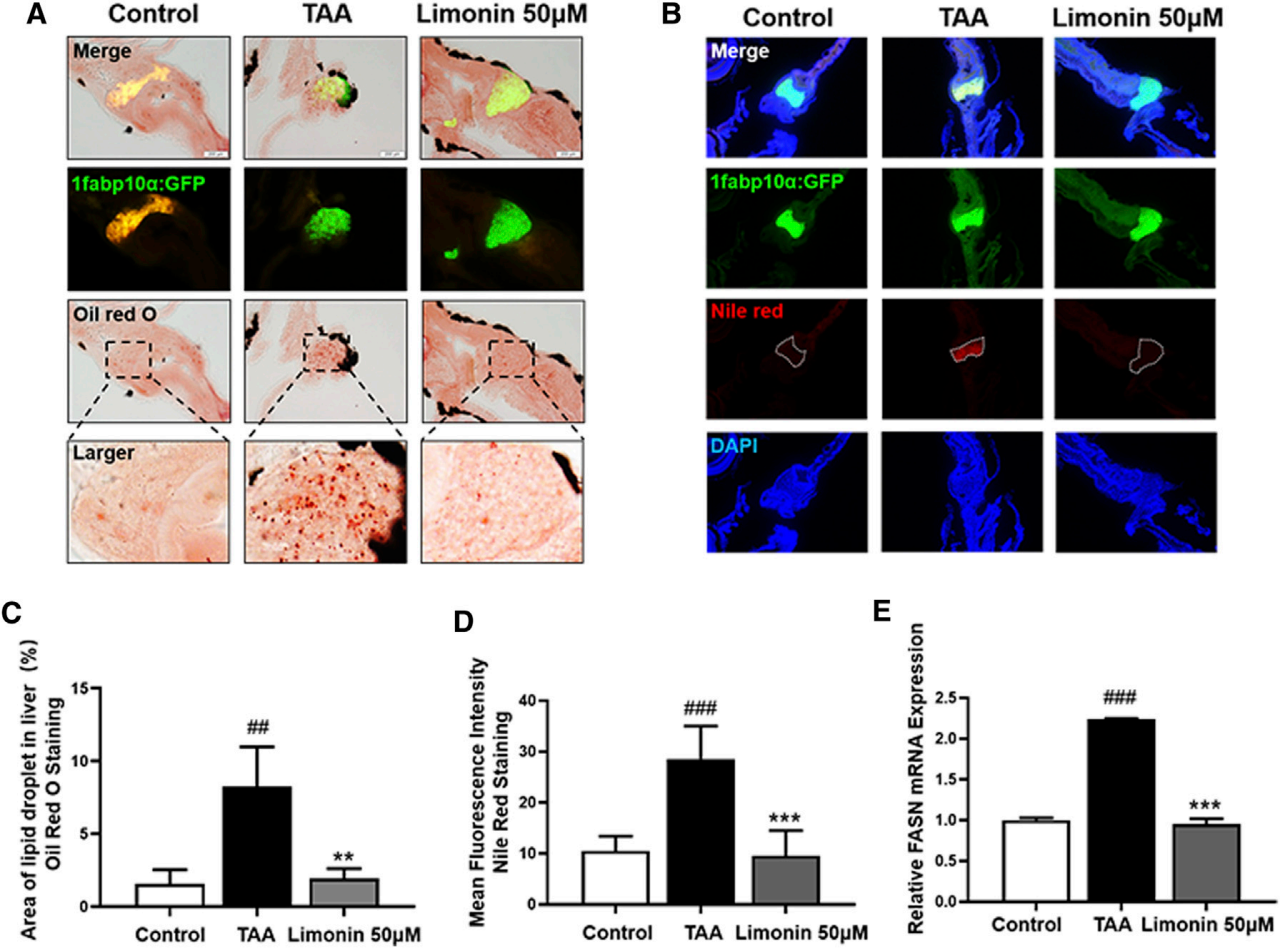
Limonin reduced lipid accumulation caused by TAA in zebrafish liver. **(A)** Oil red O staining of frozen sections of zebrafish larvae. **(B)** Frozen liver sections of zebrafish larvae with liver-specific eGFP expression were stained with Nile Red. Figures are magnified as ×200. **(C)** Quantitative analysis of the area of lipid droplet in liver based on Oil Red O staining. **(D)** Quantitative analysis of the liver mean fluorescence intensity of Nile Red. **(E)** Real-time PCR analysis of FASN mRNA levels in zebrafish larvae. The mRNA levels were normalized to β-actin mRNA levels and presented as fold change compared with the control group. Data are shown as the mean ± SD (*n* = 6–8 per group). #*p* < 0.05, ##*p* < 0.01, ###*p* < 0.001 vs control group; *p < 0.05, **p < 0.01, ***p < 0.001 vs TAA group.

Zebrafish larvae were stimulated with 0.4 mg/ml TAA for 72 h to establish the NAFLD model successfully, which manifested by hepatocytes swelling and steatosis according to previous research (Zhang et al., 2020). Based on this model, the protective effect of limonin on NAFLD was evaluated. Hepatic steatosis is the main pathological feature in the early stage of NAFLD. H&E staining showed that the liver of zebrafish larvae exposed to TAA occurred obvious lipid accumulation and ballooning degeneration, but was reduced in limonin treatment groups (Figure 3A). The results of zebrafish oil red O staining were consistent with H&E staining, which showed the area and quantity of liver lipid droplets in the limonin-treated group were significantly reduced compared with the model group (Figures 3B,C). The results showed that 12.5, 25, and 50 μM limonin could reduce lipid accumulation caused by TAA to varying degrees, and the 50 μM was the optimal concentration of limonin to alleviate hepatic steatosis. Based on the above results, the concentration of 50 μM was used to further verify the pharmacological effects of limonin on NAFLD. Frozen sections of zebrafish were then stained with Oil Red O (Figures 4A,C) and Nile Red (Figures 4B,D) to further confirm that 50 μM limonin effectively reduced lipid accumulation in the liver after TAA exposure. Fatty acid synthase (FASN) plays a key role in the initial process of lipid synthesis (Angeles and Hudkins, 2016). QPCR results showed that the expression level of FASN mRNA in the TAA exposure group was increased, and decreased after being treated with 50 μM limonin (Figure 4E). These data showed that limonin had the effect of ameliorating TAA-induced hepatic steatosis.

### Limonin Reduces Lipid Accumulation in the NAFLD Model of LO2 Cells

**FIGURE 5:**
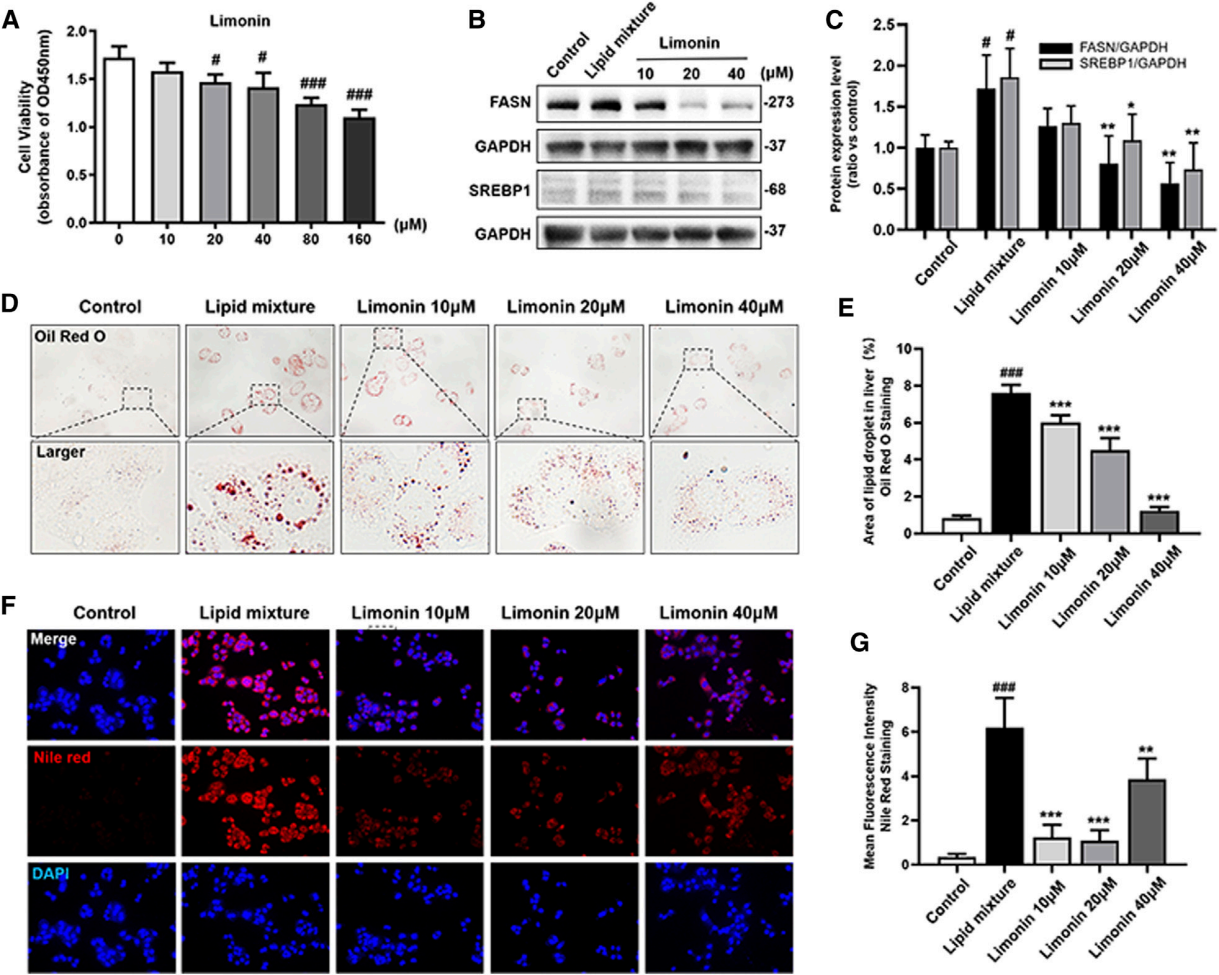
Limonin reduced lipid accumulation in LO2 cells stimulated by lipid mixture. **(A)** LO2 cells viability detection (OD 450 nm absorbance) (*n* = 4 per group). **(B)** Western blot analysis of the expression of FASN and SREBP1. GAPDH was used as an internal control. **(C)** Relative quantitative protein expression of FASN and SREBP1. **(D)** LO2 cells oil red O staining. Figures are magnified as ×400. **(E)** Quantitative analysis of the area of lipid droplets based on Oil Red O staining in LO2 cells. **(F)** Nile red staining of LO2 cells. Figures are magnified as ×200. **(G)** Quantitative analysis of the mean fluorescence intensity of Nile Red. Data are shown as the mean ± SD from three independent experiments. #*p* < 0.05, ##*p* < 0.01, ###*p* < 0.001 vs control group; *p < 0.05, **p < 0.01, ***p < 0.001 vs TAA group.

We also verified the effect of limonin on lipid accumulation in hepatocytes *in vitro*. We first established an NAFLD model of LO2 cells using lipid mixture to study the effect of limonin on lipid accumulation in hepatocytes. Based on the result of the cell viability experiment (Figure 5A), we chose 10, 20, and 40 μM limonin for the experiment. Consistent with the results of the zebrafish experiments *in vivo*, both oil red O staining (Figures 5D,E) and Nile red staining (Figures 5F,G) showed that lipid mixture stimulation significantly induced intracellular lipid accumulation, while effectively reduced in limonin-treated groups. Moreover, sterol regulatory element binding protein 1 (SREBP-1) is a membrane-bound transcription factor that participates in many functions of lipid homeostasis, especially the process of lipid synthesis (Guo et al., 2014). Results of western blot showed that the expression levels of FASN and SREBP1 in the limonin-treated group were lower than model group (Figures 5B,C). These results indicated that limonin also effectively reduced lipid accumulation in hepatocytes *in vitro*.

### Limonin Ameliorates Liver Inflammation During TAA-Induced NAFLD

**FIGURE 6:**
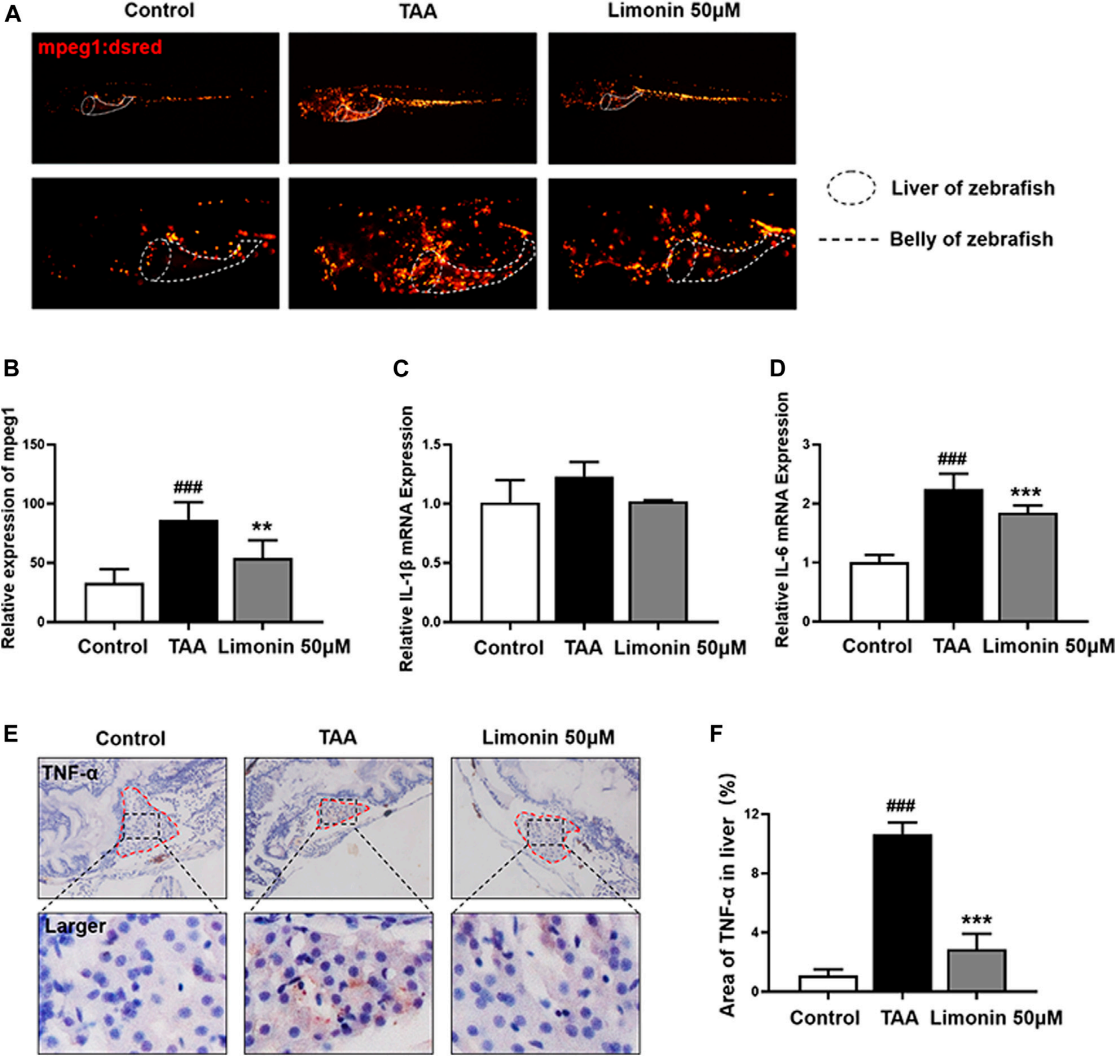
Limonin reduced liver inflammatory damage caused by TAA. **(A)** Limonin reduced the infiltration of macrophages in the liver of zebrafish during TAA exposure. Figures are magnified as ×25 and 50× (*n* = 6 per group). **(B)** Relative expression of mpeg1 in zebrafish liver. **(C,F)** Immunohistochemical staining of TNF-α and F4/80 in paraffin sections of zebrafish larvae (*n* = 4 per group). **(D,G)** Quantify the fluorescence intensity of TNF-α and F4/80 proteins. **(E,H)** Real-time PCR analysis of IL-1β and IL-6 mRNA levels in zebrafish larvae. The mRNA levels were normalized to β-actin mRNA levels and presented as fold change compared with the control group (*n* = 3 per group). Data are shown as the mean ± SD. #*p* < 0.05, ##*p* < 0.01, ###*p* < 0.001 vs control group; *p < 0.05, **p < 0.01, ***p < 0.001 vs TAA group.

A large number of experimental and clinical data support that inflammatory cells, especially macrophages, play a central role in the occurrence and development of NAFLD and NASH (Koyama and Brenner, 2017). And limonin has been reported to serve the effect of modulating immune and inflammatory responses to alleviate injury (Ishak et al., 2021). Therefore, we utilize zebrafish with transgenic strain macrophages-specific dsred-labeled to observe the distribution of macrophages in real time. We found that TAA exposure caused macrophages infiltration in liver, and 50 μM limonin could reduce the migration of macrophages to the liver (Figures 6A,B). Moreover, immunohistochemical staining showed that the expression of tumor necrosis factor α (TNF-α) and F4/80 protein increased in the TAA exposure group, but effectively decreased after 50 μM limonin treatment (Figures 6C,D, Figures 6F–G). Real-time PCR analysis also showed that compared with the control group, the TAA-treated group significantly increased the mRNA expression of Interleukin-6 (IL-6) and Interleukin-1β (IL-1β). However, these pro-inflammatory factors reduced in the limonin treatment group (Figures 6E,H). These results indicated that limonin reduced the infiltration of macrophages and the expression of inflammatory factors TNF-α, IL-1β and IL-6 secreted by macrophages in the liver, thereby ameliorating the inflammatory damage in the process of NAFLD.

### Limonin Protects Zebrafish Larvae From Oxidative Damage in NAFLD

**FIGURE 7:**
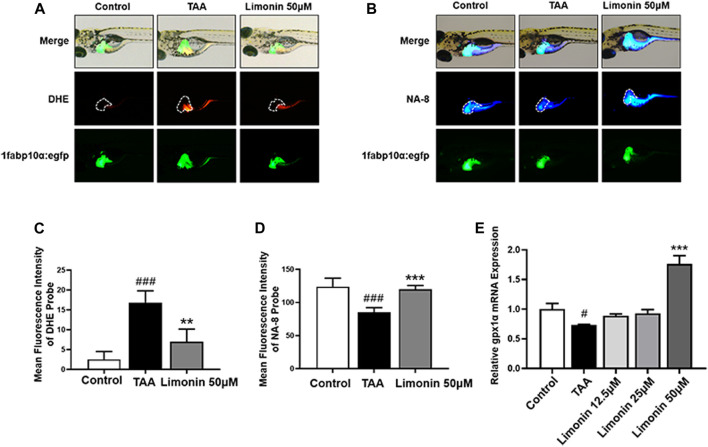
Limonin protected zebrafish larvae against oxidative stress. **(A)** Fluorescence micrographs of DHE. Figures are magnified as 50×. **(B)** Fluorescence micrographs of NA-8. Figures are magnified as ×50 **(C)** ROS quantification according to the mean intensity of red fluorescence. **(D)** GSH quantification according to the mean intensity of blue fluorescence. **(E)** Real-time PCR analysis of gpx1a mRNA levels in zebrafish larvae. The mRNA levels were normalized to β-actin mRNA levels and presented as fold change compared with the control group. Data are shown as the mean ± SD (*n* = 4–6 per group). #*p* < 0.05, ##*p* < 0.01, ###*p* < 0.001 vs control group; *p < 0.05, **p < 0.01, ***p < 0.001 vs TAA group.

Free fatty acids, which are elevated in the liver by lipid accumulation or insulin resistance, lead to incomplete oxidation in the mitochondria, peroxisomes, and microsomes, leading to the production of ROS, and subsequently accelerates the progression of NAFLD (Chen et al., 2020). Elevated levels of ROS modulate the activity of immune signaling, induce DNA damage and affect the expression and activity of key enzymes involved in lipid metabolism (Srinivas et al., 2019). GSH is a vital factor in the endogenous protection system to eliminate ROS in NAFLD (Brigelius Flohe and Maiorino, 2013). Therefore, DHE and NA-8 fluorescent probes were used to detect the distribution and levels of ROS and GSH to evaluate the TAA-induced oxidative damage in the liver of zebrafish larvae and the protective effect of limonin. The results showed strong red fluorescence appeared in zebrafish liver after TAA exposure, and 50 μM limonin could effectively reduce TAA-induced ROS accumulation (Figures 7A,C). Simultaneously, the blue fluorescence emitted by the NA-8 probe in the zebrafish liver of TAA-treated group was weakened, indicating glutathione depletion caused by TAA. However, glutathione in the liver increased after limonin treatment, which also strongly supported the protective effect of limonin on oxidative stress (Figures 7B,D). Besides, real-time PCR analysis showed that compared with the control group, the mRNA level of antioxidant gene gpx1a in model group was significantly reduced, but reversed after 50 μM limonin treatment (Figure 7E). These outcomes demonstrated that limonin played an important role in resisting oxidative stress in the progression of NAFLD induced by TAA.

### Limonin Exerts Antioxidant Effect by Enhancing NRF2 and HO-1

**FIGURE 8:**
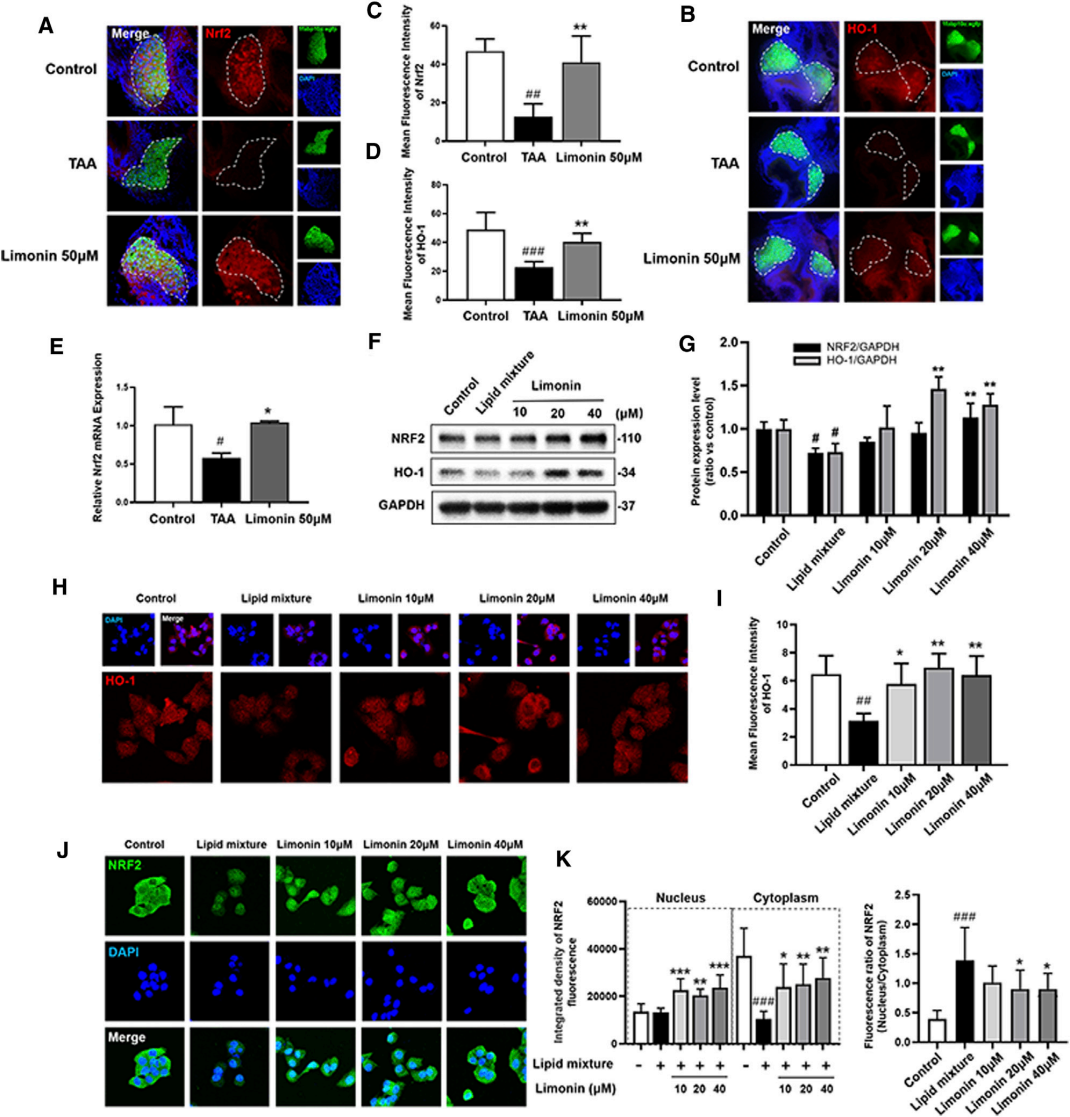
Limonin exerts the effect of anti-oxidative stress through the NRF2/HO-1 pathway. **(A)** Frozen liver sections of zebrafish larvae with liver-specific eGFP expression were immunofluorescent stained with NRF2 (*n* = 6–8 per group). **(B)** Immunofluorescence staining of HO-1 in zebrafish larvae (*n* = 6–8 per group). **(C–D)** Quantitative analysis of the liver mean fluorescence intensity of NRF2 and HO-1. **(E)** Real-time PCR analysis of NRF2 mRNA levels in zebrafish larvae. The mRNA levels were normalized to β-actin mRNA levels and presented as fold change compared with the control group (*n* = 3 per group). **(F)** Western blot analysis of the expression of HO-1 and NRF2. GAPDH was used as an internal control. **(G)** Relative quantitative protein expression of NRF2 and HO-1. **(H)** Immunofluorescence staining of HO-1 in LO2 cells. **(I)** Cellular fluorescence intensity of HO-1. **(J)** Immunofluorescence staining of NRF2 in LO2 cells. **(K)** The respective fluorescence intensity of the nucleus, cytoplasm and ratio of NRF2. Data are shown as the mean ± SD from three independent experiments. #*p* < 0.05, ##*p* < 0.01, ###*p* < 0.001 vs control group; *p < 0.05, **p < 0.01, ***p < 0.001 vs TAA group.

Transcription factor nuclear factor-erythroid 2-related factor 2 (NRF2) controls cellular adaptation to oxidants and electrophiles by inducing antioxidant genes like heme oxygenase-1 (HO-1) in response to redox stress (Hayes and Mcmahon, 2009; Villeneuve et al., 2010). The NRF2/HO-1 signaling pathway has been confirmed to play a role in inhibiting lipid accumulation and oxidative stress in NAFLD (Shen et al., 2019). In our study, immunofluorescence staining and real-time quantitative PCR showed that compared with the control group, the expression of NRF2 and HO-1 proteins were down-regulated after TAA exposure, whereas increased with limonin treatment (Figures 8A–E). Similarly, the LO2 cells experiments showed that limonin could reverse the decrease of NRF2/HO-1 expressions caused by TAA (Figures 8F–I). Meanwhile, we also detected the distribution of NRF2 in LO2 cells. NRF2 was mainly located in the cytoplasm in normal cells, and lipid mixture could partially transfer NRF2 from the cytoplasm to the nucleus. Moreover, limonin treatment significantly promoted the nuclear expression of Nrf2 (Figures 8J,K). Overall, these results supported the important role of limonin-mediated antioxidant capacity in the development of NAFLD.

## Discussion

As the rising prevalence has caused an increasing economic burden, and with more and more patients with liver cirrhosis and end-stage liver disease requiring liver transplantation, NAFLD has become a disease of major concern (Friedman et al., 2018). However, the pathogenesis of NAFLD has not yet been fully elucidated, and the determination of therapeutic targets and the advancement of drug development are severely limited. Current research shows that limonin has a wide range of pharmacological effects, including anti-cancer, anti-inflammation, anti-bacterial, anti-viral, anti-oxidation and liver protection properties (Fan et al., 2019), and has been proven to have the effect of preventing and treating alcoholic fatty liver (Valansa et al., 2020) and various acute liver injuries (Mahmoud et al., 2014a; Mahmoud et al., 2014b; Yang et al., 2021). But the therapeutic potential of limonin in NAFLD is still unknown. In this study, we clarified that limonin could alleviate fatty liver disease *in vitro* and *in vivo* experiments, including decreased liver lipid accumulation and down-regulated the expression of lipogenic transcription factors FASN and SREBP1, which were up-regulated by *de novo* lipogenesis and ultimately induce steatosis (Anderson and Borlak, 2008).

Researchers have proposed the “multiple hits” theory to explain the pathogenesis of NAFLD (Buzzetti et al., 2016). It is believed that various subsequent injuries such as inflammatory infiltration and oxidative stress can accelerate the development of NAFLD. Macrophages are the major cells that respond to liver injury and are responsible for triggering tissue inflammation, through phagocytosis and the production of cytokine/growth factors including TNF-α, IL-6 and IL-1β(Acuner et al., 2014; Landskron et al., 2014; Kwon et al., 2016; Liu et al., 2017). And obesity and cytokine factors have been shown to be linked by certain stimulating factors like stem cell growth factor-beta (Tarantino et al., 2020). In this study, we used macrophage-labeled zebrafish to observe the distribution of macrophages in real time and found that limonin reduced TAA-induced recruitment of macrophages in the liver. F4/80 is a cell surface glycoprotein that specifically labels macrophages. Compared with model group, the expression of F4/80 in the limonin treatment group was significantly reduced. Moreover, the expression levels of pro-inflammatory factors such as TNF-α, IL-6 and IL-1β secreted by macrophages in the liver of zebrafish exposed to TAA were increased, but reduced after limonin treatment. These results indicated that limonin could prevent TAA from causing further inflammatory damage to the liver.

Studies have shown that increased infiltration of pro-inflammatory macrophages stimulated by lipid accumulation could produce a large amount of ROS through dynein-mediated endocytosis, which participates in the development of NAFLD (Kim et al., 2017). The unrestricted production of free radicals and ROS can cause damage to important biomolecules and cells. And GSH is a significant factor in the endogenous protection system for eliminating ROS(Brigelius-Flohe and Maiorino, 2013). Therefore, we used DHE and NA-8 probes to detect zebrafish liver ROS and GSH levels. After TAA stimulation, the liver ROS levels increased significantly and GSH was severely depleted. But limonin treatment reduced the accumulation of ROS and partially restored GSH. Besides, gpx1a has been confirmed to have an antioxidant effect in NAFLD (Wang et al., 2020). Our results showed that limonin could reverse the decrease in gpx1a caused by TAA. These results indicated that limonin had the effect on resisting oxidative damage.

NRF2 is a nuclear transcription factor related to the defense against oxidative stress damage. It can reduce liver damage by regulating the expression of a variety of cytoprotective enzymes, including HO-1 (Tang et al., 2014). Studies have proved that limonin could inhibit inflammation by activating the Nrf2 antioxidant pathway to improve drug-induced liver injury (Yang et al., 2020). Our results indicated that the whole cells expression of NRF2 and HO-1 in the model group of zebrafish and LO2 were decreased, while effectively increased in limonin treatment groups. Meanwhile, NRF2 is a stress-sensitive genetic transcription factor that appears to be a major regulator of cell response to oxidative damage and other stress conditions. In the state of inflammation or stress injury, NRF2 spontaneously undergoes nuclear translocation to resist oxidative stress and maintain cell homeostasis (Ge et al., 2017). As expected in our study, NRF2 was mainly distributed in the cytoplasm by immunofluorescence localization. Lipid mixture treatment increased the ratio of nuclear to cytoplasmic, but the expression of NRF2 in the nucleus and cytoplasm were down-regulated. And limonin induced the transfer of Nrf2 to the nucleus. These results were consistent with previously reported that limonin promoted NRF2 into nucleus to exert the anti-oxidation effect (Yang et al., 2020). All the data implied that limonin had the effect of improving the antioxidant capacity in NAFLD.

## Conclusion

**FIGURE 9:**
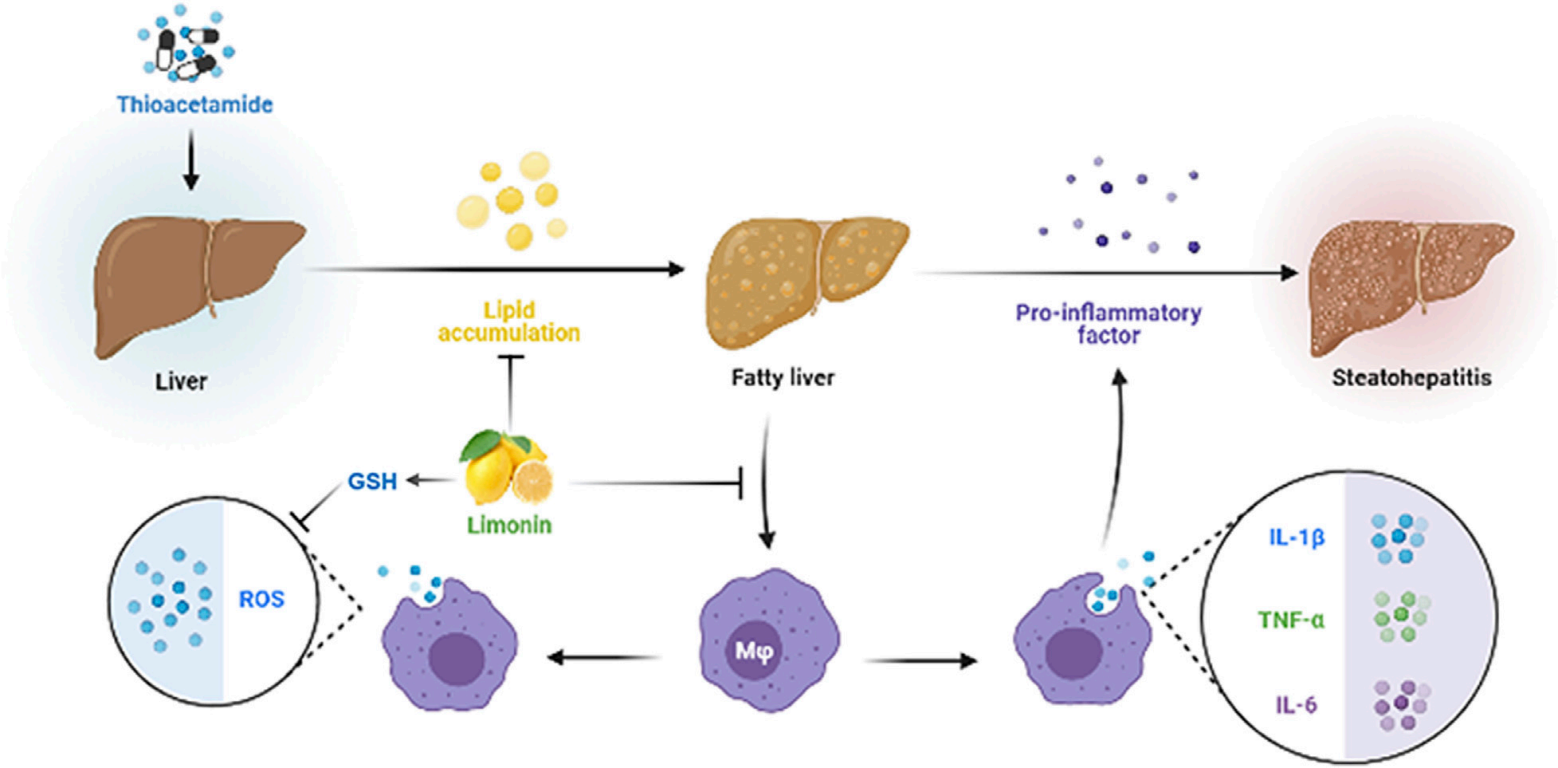
Diagram of the protective mechanism of limonin on non-alcoholic fatty liver disease.

In summary, our research revealed the pharmacological effects of limonin in the treatment of NAFLD by reducing lipid accumulation, suppressing oxidative stress and alleviating inflammation caused by pro-inflammatory chemokines and macrophages infiltration (Figure 9), providing a scientific basis for the clinical application of limonin. However, how limonin regulates lipid metabolism and interferes with oxidative stress remains unclear. It is necessary to further modify genes related to lipid metabolism and oxidative stress to clarify the specific mechanism of limonin on NAFLD.

## Data Availability

The original contributions presented in the study are included in the article/Supplementary Material, further inquiries can be directed to the corresponding authors.

## Abbreviations

NAFLD, non-alcoholic fatty liver disease; NASH, non-alcoholic steatohepatitis; ROS, reactive oxygen species; IL-6, interleukin-6; IL-1β, interleukin-1β; TNF-α, tumor necrosis factor α; FASN, fatty acid synthase; SREBP1, sterol regulatory element binding protein 1; NRF2, nuclear factor-erythroid 2-related factor 2; HO-1, heme oxygenase-1; GSH, glutathione; qPCR, quantitative polymerase chain reaction; GAPDH, glyceraldehyde-3-phosphate dehydrogenase; DMSO, dimethyl sulfoxide; dpf, days post fertilization; DMEM, dulbecco’s modified eagle medium; TAA, thioacetamide; DHE, dihydroethidium; PFA, paraformaldehyde; PBS, phosphate buffered saline; SD, standard deviation; RIPA, radio Immunoprecipitation Assay; H&E, hematoxylin and eosin; OCT, optimal cutting temperature compound; H_2_O_2_, hydrogen peroxide; DAB, diaminobenzidine tetrahydrochloride; OD, optical density; TUNEL, terminal deoxynucleotidyl transferase-mediated dUTP-biotin nick end labeling; CCK-8, cell counting kit-8; ANOVA, one-way analysis of variance.
